# Myocardial inflammatory cells in cardiac amyloidosis

**DOI:** 10.1038/s41598-024-74289-5

**Published:** 2024-10-07

**Authors:** Philip Simon, Hans-Michael Behrens, Arnt Kristen, Christoph Röcken

**Affiliations:** 1 Department of Pathology, University Hospital Schleswig-Holstein, Christian-Albrechts-University, Arnold-Heller-Str. 3, Building U33, 24105 Kiel, Germany; 2 Department of Cardiology, Angiology, Respiratory Medicine, Medical University of Heidelberg, Heidelberg, Germany

**Keywords:** Amyloid, Amyloidosis, Complement 9, Caspase 3, Cell death, Inflammation, Biomarkers, Translational research

## Abstract

Background: Immunoglobulin derived AL amyloidosis and transthyretin derived ATTR amyloidosis are the most common forms of cardiac amyloidosis. Both may present with cardiac arrhythmias, heart failure, and extracardiac symptoms. Disease outcome is often fatal. Recently, it was proposed that amyloid may cause cardiac inflammation. Here we tested the hypothesis that immune cell infiltration in cardiac tissue correlates with clinicopathological patient characteristics. Patients and methods: Myocardial biopsies from 157 patients with cardiac amyloidosis (46.5% AL, 53.3% ATTR) were immunohistochemically assessed for the presence and amount of T lymphocytes (CD3), macrophages (CD68) and neutrophils (MPO). Amyloid load, cardiomyocyte diameter, apoptosis (Caspase 3), necrosis (complement 9), and various clinical parameters were assessed and correlated with immune cell density. Results: Myocardial tissue was infiltrated with T lymphocytes (CD3), macrophages (CD68) and neutrophils (MPO) with variable amounts. Significant correlations were found between the number of macrophages and NYHA class. No correlations were found between the presence and amount of T lymphocytes, neutrophils and clinicopathological patient characteristics. Conclusion: The significant correlation between cardiac macrophage density and heart failure points towards a significant role of macrophages in disease pathology.

**Supplementary Information:**

The online version contains supplementary material available at 10.1038/s41598-024-74289-5.

## Introduction

Cardiac amyloidosis is caused by the deposition of amyloid in the extracellular space of the myocardium^1,2^ and may present with cardiac arrhythmias, heart failure and also extracardiac symptoms. While several studies have documented the prevalence of systemic AL amyloidosis, ATTRwt amyloidosis, and ATTR variant forms^3–5^, comprehensive epidemiological data covering all forms and subtypes, especially in diverse populations, remains incomplete. This gap in data contributes to challenges in understanding the full spectrum of disease morbidity and mortality. The two most common types of cardiac amyloidosis are immunoglobulin light chain-associated amyloidosis (AL) and transthyretin-derived amyloidosis (ATTR).

N-terminal pro-brain natriuretic peptide (NT-proBNP) is highly valuable for monitoring the severity and assessing the prognosis of cardiac amyloidosis. NT-proBNP is not specific to amyloidosis alone, as elevated levels can also occur in other cardiac conditions^6^. Therefore, while NT-proBNP levels can be indicative of disease severity and correlate with outcomes and survival, they should not be used in isolation for diagnosing cardiac amyloidosis.

The New York Heart Association (NYHA) functional class and cardiac amyloid load correlate with disease-related mortality^7^. Treatment of AL amyloidosis entails autologous stem cell transplantation, chemotherapy, or immunomodulatory therapy that inhibits proinflammatory cytokines and activates the innate immune response. Cardiac ATTR amyloidosis may be treated with Tafamidis, a stabilizer of the TTR tetramer. More recently advancements in the treatment of ATTR amyloidosis have been achieved, particularly by the introduction of TTR silencers such as siRNA inhibitors which expand therapeutic options, including variant and wild-type ATTR amyloidosis, as demonstrated in recent clinical trials^13^. The growing recognition of the prevalence of both AL and ATTR amyloidosis underscores the need for continued research into targeted therapies and early diagnosis to improve patient outcomes^14,15^.

The precise mechanism by which the amyloid fibrils impact on cardiac morphology and function are not entirely clear, but a combination of cellular stress, cytotoxicity, oxidative damage, and apoptosis contribute to the pathophysiology of the disease^8–10^. In an animal model of AL, injected human AL proteins caused cardiac dysfunction, induced cell death and increased mortality^11^. Subjecting cardiomyocytes to AL proteins results in the generation of reactive oxygen species, impairs contractility and causes apoptosis^8,12^.

In recent studies, the role of immune cell infiltration in heart failure was highlighted. Cardiac amyloidosis-induced heart failure is characterized by diastolic dysfunction and a preserved ejection fraction, namely heart failure with preserved ejection fraction. Patients with this condition exhibit an inflammatory cardiac state with elevation of CD3-positive T-cells, CD68-positive macrophages, and pro-inflammatory cytokines such as tumor necrosis factor-alfa (TNF-alfa) and interleukins 1beta, 6 and 10^16–19^. Infiltrating immune cells in the myocardium of patients with cardiac amyloidosis have not been characterized to date. The aim of the present study was therefore to assess the immune landscape of the myocardium in cardiac amyloidosis and correlate it with disease type, amyloid load, apoptosis, and the NYHA functional class. We hypothesize that the presence of immune cells in the myocardium is associated with the clinical and cytological features of cardiac amyloidosis.

## Materials and methods

### Ethics statement

This project was approved by the local ethics committee of the University Hospital in Kiel (D581-585/15; D 469/18) and the Medical Faculty of the University of Heidelberg (S-093/2014) conforming to the Declaration of Helsinki. Informed consent was obtained from all subjects and/or their legal guardian(s) for scientific use of access tissue specimens.

### Patients

From the Amyloid Registry of the Department of Pathology, Christian-Albrecht-University Kiel, we retrieved a series of patients with histologically confirmed cardiac amyloidosis. The samples were obtained from 2007 to 2014 and included 2 different types of amyloid, i.e., AL and ATTR. The AL amyloid of was further subdivided into ALλ and ALκ. The specimens were obtained from heart tissue and were randomly selected. The only inclusion criterion was the histologically confirmed presence of amyloid.

The study cohort enclosed specimens from 157 patients [128 men (81.5%) and 29 woman (18.5%); median age 69.76 years] (Table 1). 84 patients suffered from ATTR (median age 72.07 years; range 53.3–86.5 years), 73 from AL amyloidosis (median age 64.7 years; range 37.0–86.0 years), including 62 from ALλ amyloidosis (median age 63.5; range 37.0–86.0) and 11 from ALκ amyloidosis (median age 65.3; range 52.9–80.2).

**Table 1:** Patient characteristics, clinical and histological parameters. AL: light chain amyloidosis; ALλ: light chain lambda amyloidosis; ALκ: light chain kappa amyloidosis; ATTR: transthyretin amyloidosis; LA: left atrial; NYHA: New York Heart Association; NTproBNP: n-terminal pro brain natriuretic peptide; MAPSE: mitral annular plane systolic excursion; LA size: left atrial size.

		All cases	AL (all)	ALλ	ALκ	ATTR
		*n* (%)	*n* (%)	*n* (%)	*n* (%)	*n* (%)
**Parameter**	**Total**	157	73	62	11	84
**Gender**	**n/missing**	157/0	73/0	62/0	11/0	84/0
Male		128 (81.5)	51 (69.9)	18 (29.0)	4 (36.4)	77 (91.7)
Female		29 (18.5)	22 (30.1)	44 (71.0)	7 (63.6)	7 (8.3)
**Age**	**n/missing**	147/0	73/0	62/0	11/0	84/0
Median [min - max]		69.8 [37.0–86.5]	64.7 [37.0–86.0]	63.5 [37.0–86.0]	65.3 [52.9–80.2]	72.1 [53.3–86.5]
**NYHA**	**n/missing**	151/6	70/3	59/3	11/0	81/3
1		3 (2.0)	1 (1.4)	1 (1.7)	0 (0.0)	2 (2.5)
2		49 (32.5)	22 (31.4)	17 (28.8)	5 (45.5)	27 (33.3)
3		96 (63.6)	46 (65.7)	40 (67.8)	6 (54.5)	50 (61.7)
4		3 (2.0)	1 (1.4)	1 (1.7)	0 (0.0)	2 (2.5)
**NT-proBNP [ng/ml]**	**n/missing**	150/7	72/1	61/1	11/0	78/6
Median [Q1/Q3]		5893 [2553/11060]	8119 [5058/4865]	8152 [5259/14252]	6820 [3461/21140]	3683 [1740/7631]
**LA size [mm]**	**n/missing**	149/7	72/1	61/1	11/0	77/7
Median [Q1/Q3]		44 [41/48]	44 [40/48]	44 [40/48]	42 [46/48]	44 [41/47]
**Mitral annular plane systolic excursion (MAPSE) [m/s]**	**n/missing**	148/9	71/2	62/0	9/2	77/7
Median [Q1/Q3]		0.7 [0.6/0.98]	0.6 [0.5/0.9]	0.6 [0.5/0.9]	0.7 [0.6/0.8]	0.8 [0.6/1.0]
**Precordial voltage**	**n/missing**	142/15	68/5	58/4	10/1	74/10
Median [Q1/Q3]		15.5 [11.8/20.0]	16.0 [13.0/20.0]	16.0 [13.0/20.0]	16.0 [12.8/21.0]	15.0 [8.8/19.3]
**Limb voltage**	**n/missing**	142/15	68/5	58/4	10/1	74/10
Median [Q1/Q3]		7.0 [5.0/10.0]	7.0 [5.0/9.0]	7.0 [5.0/9.0]	6.0 [4.0/8.3]	7.0 [3.0/11.5]
**Diameter of cardiomyocytes [µm]**	**n/missing**	126/31	60/13	51/11	9/2	66/18
Median [Q1/Q3]		21.5 [19.2/26.1]	21.3 [18.7/26.3]	20.8 [18.6/26.3]	23.1 [19.0/29.9]	21.9 [19.8/26.0]
**Presence of distinct C9 positive myocytes**	**n/missing**	99/58	50/23	43/19	7/4	49/35
Absent		83 (83.8)	36 (72.0)	32 (74.4)	4 (57.1)	47 (95.9)
Present		16 (16.2)	14 (28.0)	11 (25.6)	3 (42.9)	2 (4.1)
**Proportion of C9 positive area [%]**	**n/missing**	75/82	37/36	31/31	6/5	38/46
Median [Q1/Q3]		6.0 [2.1/12.9]	2.9 [1.2/7.7]	2.9 [1.2/6.2]	7.8 [1.4/16.4]	10.1 [4.0/14.9]
**Proportion of Cas3 positive area [%]**	**n/missing**	89/68	47/26	40/22	7/4	42/42
Median [Q1/Q3]		0.48 [0.15/2.05]	0.24 [0.07/0.52]	0.24 [0.06/0.49]	0.52 [0.11/2.06]	1.21 [0.31/5.16]
**Density of CD3 positive cells [n/mm** ^**2**^ **]**	**n/missing**	89/68	44/29	37/25	7/4	45/39
Median [Q1/Q3]		0.0 [0.0/0.47]	0.13 [0.0/0.61]	0.08 [0.0/0.62]	0.28 [0.0/0.54]	0.0 [0.0/0.20]
**Density of CD68 positive cells [n/mm** ^**2**^ **]**	**n/missing**	58/99	32/41	27/35	5/6	26/58
Median [Q1/Q3]		0.0 [0.0/1.01]	0.0 [0.0/1.32]	0.0 [0.0/1.35]	0.49 [0.0/1.62]	0.0 [0.0/0.80]
**Density of MPO positive cells [n/mm** ^**2**^ **]**	**n/missing**	52/105	30/43	27/35	3/8	22/62
Median [Q1/Q3]		0.21 [0.0/0.73]	0.23 [0.0/0.57]	0.22 [0.0/0.51]	0.41 [0.0/0.41]	0.18 [0.0/1.15]

### Clinical patient characteristics

The study cohort was part of a previous study^7^. In brief, patient records were analyzed for standard blood tests, 12-lead electrocardiography, and echocardiography. Cardiac troponin T was either measured by the fourth-generation assay or by the high-sensitivity assay (Roche Diagnostics, Mannheim, Germany). Due to lack of comparability of the different troponin assays abnormal values were defined as > 0.03 µg/L (fourth-generation assay) and > 14 pg/ml (high-sensitivity assay). NT-proBNP was measured using Elecsys proBNP (Roche Diagnostics).

Electrocardiography was analyzed for low voltage pattern defined as QRS complex deflection below 0.5 mV in any limb leads or the sum of the S-wave deflection in V1-2 and R-wave deflection in V5–6 was < 1.5 mV. Transthoracic echocardiograms were analyzed for surrogate markers of cardiac amyloidosis, e.g. left atrial size (LA size) and mitral annular plane systolic excursion (MAPSE). LA size was calculated as reported previously^20^. MAPSE was obtained from the apical four-chamber view using M-mode imaging^21^.

### Histology

All samples were fixed in formalin, embedded in paraffin, and stained with hematoxylin and eosin (H&E) as well as Congo red. The presence of amyloid was verified by observing characteristic birefringence under a polarization microscope. Further details are documented in the Supplementary Methods File.

### Immunohistochemistry

Immunohistochemical analysis was performed using monoclonal and polyclonal antibodies directed against complement 9, CD68, caspase 3, anti-λ-light chain peptides, transthyretin, CD3 and myeloperoxidase to identify amyloid proteins and associated cellular markers. Details of the antibodies used, antigen retrieval methods, and staining procedures are fully documented in the Supplementary Methods File.

### Evaluation of immunostaining

Serial sections were used throughout this study. Initially, we confirmed the presence of amyloid in the first serial section. Next, the distribution of amyloid deposits was compared with the immunostaining of the putative amyloid proteins. Then, the spatial distribution of the classified amyloid proteins was compared to the spatial distribution of the Cas3- and C9 immunostaining. The intensity of immunostaining was graded as absent (0), weak (1+), moderate (2+) or strong (3+). A staining intensity of 1 + to 3 + was considered positive. The percentage of the target proteins detected within the amyloid deposits was documented in steps of 5%. In addition, cellular immunostaining of both Cas3 and C9 was documented in relation to the amyloid deposits. In order to provide an impression of the extent of immunolabeling, we evaluated the percentage area of amyloid deposits stained with antibodies directed against C9 and Cas3.

### Quantification of amyloid load

The amyloid load of a tissue section was calculated by dividing an area containing amyloid deposits by the total tissue area, resulting in a ratio of 0 to 100%. To determine both areas, Congo red stained tissue slides were scanned in consecutive bright field and fluorescence mode using a Hamamatsu NanoZoomer 2.0 RS scanner (Hamamatsu Photonics Deutschland GmbH, Herrsching am Ammersee, Germany) with fluorescence option. The bright field image was used to count the pixels comprising total tissue by applying the color threshold function of ImageJ, version 1.52p^24^ and adjusting brightness and saturation values until all tissue areas were selected. Fluorescence of Congo red was visualized in the Alexa Fluor 555 channel with an excitation wavelength of 560 nm and an emission wavelength of 607 nm, appearing yellow orange. This channel also showed an undesirable diffuse autofluorecence of the whole tissue. To compensate for this, tissue sections were also scanned in the Alexa Fluor 488 channel with excitation wavelength of 485 nm and emission wavelength of 525 nm, appearing green. This channel showed only autofluorescence of the tissue, but no signal from Congo red. In the overlay of both images, Congo red appeared yellow orange to orange, while background and nonamyloid tissue appeared green to yellow green. The color threshold function of ImageJ was used to select all pixels showing amyloid, this time by adjusting the hue value. Sections were scanned at 400x magnification and images analyzed with ImageJ.

### Quantification of immune cells

The number of CD3-, MPO-, and CD68-positive cells within the selected area was determined using the image analysis software Definiens Tissue Studio Version 4.4.2 (Definiens AG, Munich, Germany) in the cell marking mode. Subsequently, a measurement of the selected tissue area was performed, typically corresponding to the entire available tissue section. To determine the density of immunostained immune cells in the tissue, the ratio of immune cells to pixels of the entire selected tissue area was calculated.

### Measurement of cardiomyocyte diameter

Cardiomyocyte diameter was measured on H&E-stained tissue sections using a Nikon Eclipse microscope (Nikon, Minato, Japan) equipped with a digital camera. An image of a representative area was captured at 400x magnification using Nikon software. In the captured image, 30 cardiomyocytes were selected, and their diameter measured using the ruler tool in a way that best represented the perpendicular crosscut of the cylindric cardiomyocytes, i.e., taking the smallest diameter when the cardiomyocyte cross-cut was elliptical. The mean value was then used for further analysis.

### Statistics

Statistical analyses were done using SPSS 27.0 (IBM Corporation, Armonk, New York, USA). The significances of associations between non-ordinal variables were calculated using Fisher´s exact test. Kendall´s tau test was applied for parameters of ordinal scale (NYHA). We assumed a significance level of 0.05. To compensate for the false discovery rate within the correlations, we applied the Simes (Benjamini-Hochberg) procedure (multiple testing correction). All p-values are shown uncorrected. The p-values, which remained significant after the Simes procedure, are marked in Table 2.

**Table 2:** Correlation between CD68 positive cells and clinical parameters. AL: light chain amyloidosis; ATTR: transthyretin amyloidosis; CD68: cluster of differentiation 68; LA: left atrial; NYHA: New York Heart Association; NTproBNP: n-terminal pro brain natriuretic peptide. (a) significant after false discovery rate-correction.

Parameter	Density of CD68 positive cells, all cases			Density of CD68 positive cells, AL only			Density of CD68 positive cells, ATTR only		
	Low	High	*p*-value	Low	High	*p*-value	Low	High	*p*-value
	*n* (%)	*n* (%)		*n* (%)	*n* (%)		*n* (%)	*n* (%)	
**NYHA low/high**									
**1/2**	13 (86.7)	2 (13.3)	**0.006** ^**a**^	7 (87.5)	1 (12.5)	**0.041** ^**a**^	6 (85.7)	1 (14.3)	**0.090**
**3/4**	18 (42.9)	24 (57.1)		10 (41.7)	14 (58.3)		8 (44.4)	10 (55.6)	
**NTproBNP**									
**Low**	15 (71.4)	6 (28.6)	**0.054**	5 (62.5)	3 (37.5)	**0.049** ^**a**^	10 (76.9)	3 (23.1)	**0.038** ^**a**^
**High**	15 (42.9)	20 (57.1)		12 (50.0)	12 (50.0)		3 (27.3)	8 (72.7)	

## Results

### Histology

**Fig. 1:**
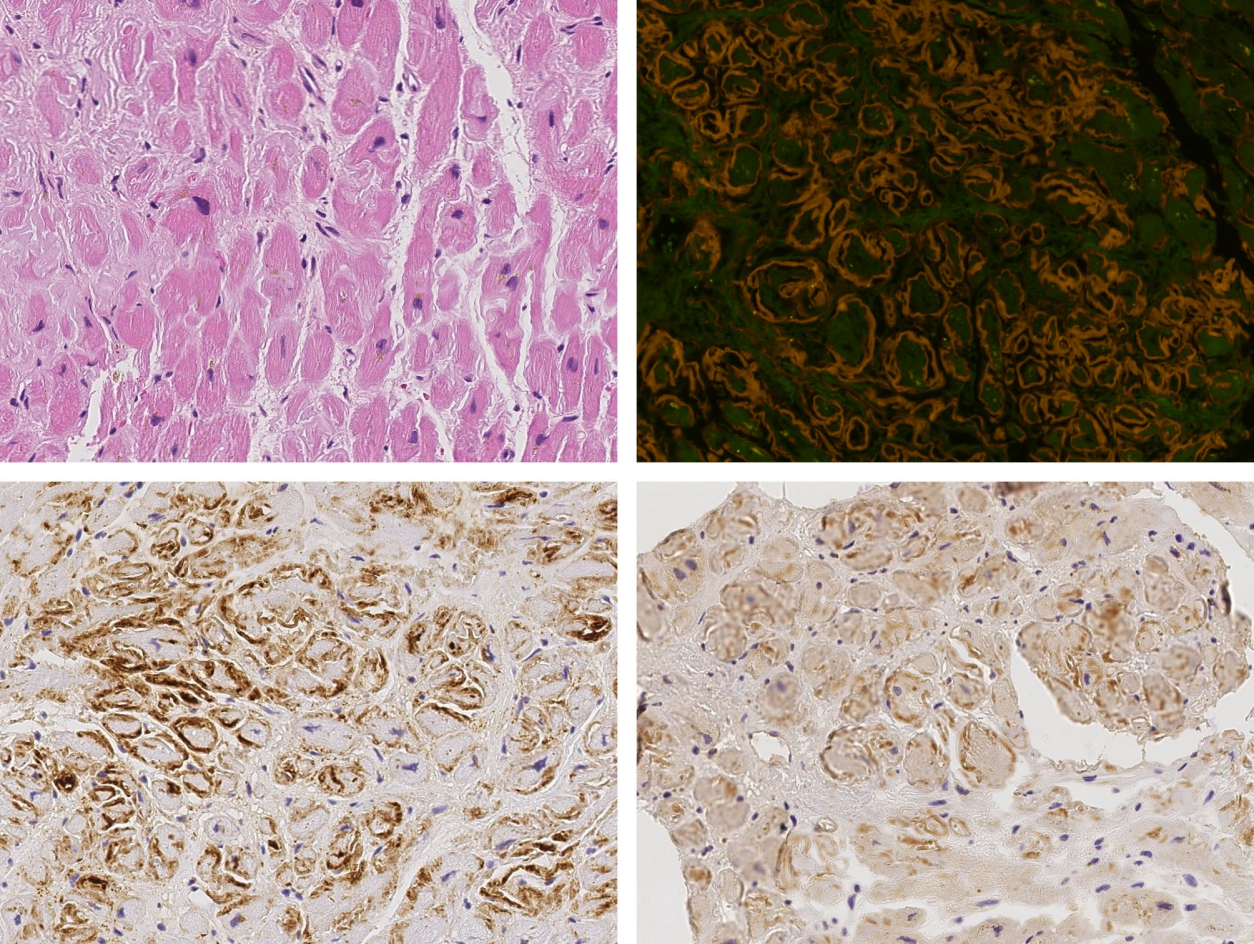
Cardiac amyloidosis, single cell necrosis and apoptosis. Comparison between hematoxylin and eosin staining (**A**), Congo red staining in fluorescence microscopy (**B**), immunostaining of amyloid with an anti-complement 9 antibody (**C**) and an anti-caspase 3-antibody (**D**). Original magnifications 200-fold, hematoxylin counterstain (**C,D**).

The presence of amyloid was confirmed histologically in every tissue sample using Congo red staining and polarization microscopy (Fig. 1). The median total size of the tissue samples was 3.9 mm^2^ (range 0.08–39.3 mm^2^). The median amyloid load was 44.7% [range: 1.2–86.9%; ATTR: 43.1% (range:1.7–86.9%); AL: 45.4% (range: 1.2–83.9%)]. Amyloid load was not significantly different between ATTR and AL amyloid.

The medium diameter of cardiomyocytes in cardiac amyloidosis was 21.5 μm (range 13.0–37.0 μm). There was no significant difference between ATTR- and AL amyloidosis (*p* = 0.859) and no correlation with amyloid load (*p* = 1.000).

### Immunostaining of complement 9 and caspase 3

First, we examined the presence of C9 and Cas3 in cardiac amyloidosis. C9 was assessable in 81 cases, Cas3 in 89 cases (Fig. 1).

C9 immunolabeling in heart tissue was noted in all cases (81 cases). We observed in AL and ATTR amyloid cases that antibodies directed against C9 matched amyloid deposits in all cases homogeneously. Interstitial immunostaining of C9 was not present outside the amyloid deposits in any case confirming findings made previously by Lux et al.^25^.

Cas3 immunostaining of the amyloid deposits was also noted in all cases and the percentage area of amyloid deposits stained with Cas3 varied from 0.2 to 15.5%. There was no significant correlation between amyloid load and Cas3 positive tissue.

Subsequently, we examined the cellular expression of C9 in cardiomyocytes (i.e., “C9 positive myocytes”). In total, 16 out of 81 available (19.75%) cases were found to enclose any C9 positive single cells. The presence of C9 positive myocytes did not correlate with amyloid load in all cases, cases with AL only or cases with ATTR only, respectively.

### Inflammatory cells

**Fig. 2:**
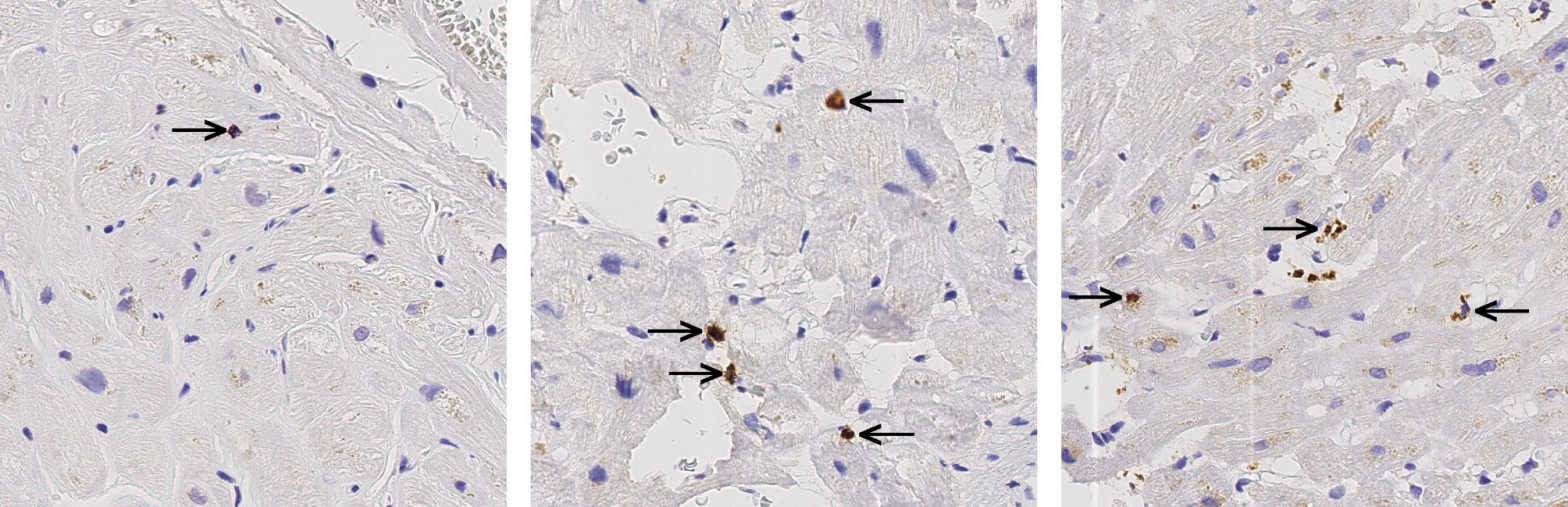
Inflammatory cells in the myocardium of cardiac amyloidosis. Exemplary immunostaining with antibodies directed against CD3 (T lymphocytes) (**A**), myeloperoxidase (neutrophiles) (**B**), CD68 (macrophages) (**C**) on an area of 0.12 mm^2^. Arrows show positive corresponding immune cells (1 T lymphocyte, 4 neutrophils and 3 macrophages). Original magnifications 400-fold, hematoxylin counterstain.

Next, we performed immunostaining for T lymphocytes (CD3), neutrophils (MPO) and macrophages (CD68) (Fig. 2). 86 cases were available for CD3-, 57 for CD68- and 50 for MPO immunostaining. CD3 positive T lymphocytes were found in 38 (44.2%) cases, MPO positive polymorphonucleocytes in 31 (62.0%) cases and CD68 positive macrophages in 27 (47. %) cases. Thereafter we evaluated the density of immune cells. The highest density was observed in MPO positive cells (0–31.9 per mm^2^; median 0.21 per mm^2^). Neither CD3 positive cells nor MPO positive cells nor CD68 positive cells correlated significantly with amyloid load. A separate analysis of AL- and ATTR cases also did not show significant correlations between amyloid load and CD68 density.

In the next step we examined the correlation between clinical parameters and amyloid load in AL amyloid only, ATTR amyloid only, all amyloid cases, the diameter of cardiomyocytes and the density of immune cells. The clinical parameters included New York Heart Association class (NYHA class), N-terminal pro brain natriuretic peptide (NTproBNP), left atrial size, mitral annular plane systolic excursion, precordial voltage and limb voltage. A significant correlation was found in AL cases only between amyloid load and NYHA class (*p* = 0.007 for NYHA 1 to 4 and *p* < 0.05 for NYHA low/high), which lost significance after correction for multiple testing. In ATTR cases only, and in all cases there was no significant correlation between amyloid load and clinical patient characteristics. A correlation was also found between cardiomyocyte diameter and TDIs (*p* = 0.024), which lost significance after correction for multiple testing. Cardiomyocyte diameter did not correlate with NYHA (*p* = 0.702), NTproBNP (*p* = 0.368), LA size (*p* = 0.153), MAPSE (*p* = 0.856), or voltages (precordial: *p* = 0.099, limb: *p* = 0.343). Neither CD3 positive cells nor MPO positive cells correlated with any of the clinical patient characteristics. For CD68 positive cells, significant correlations were found with the NYHA classes 1 to 4. In general, the number of CD68 positive macrophages was significantly higher in advanced NYHA stages (all cases and AL cases) (Table 2; significant after correction for multiple testing). An insignificant finding was made in ATTR (Table 2). Elevated NTproBNP levels corresponded with higher CD68 cell numbers across all cases, while the correlation did not reach statistical significance after correction for multiple testing in the groups AL (*p* = 0.049) and ATTR (*p* = 0.038) (Table 2). CD68 cell density did not correlate with LA size, TDIs, precordial voltage, limb voltage, or MAPSE.

## Discussion

**Fig. 3:**
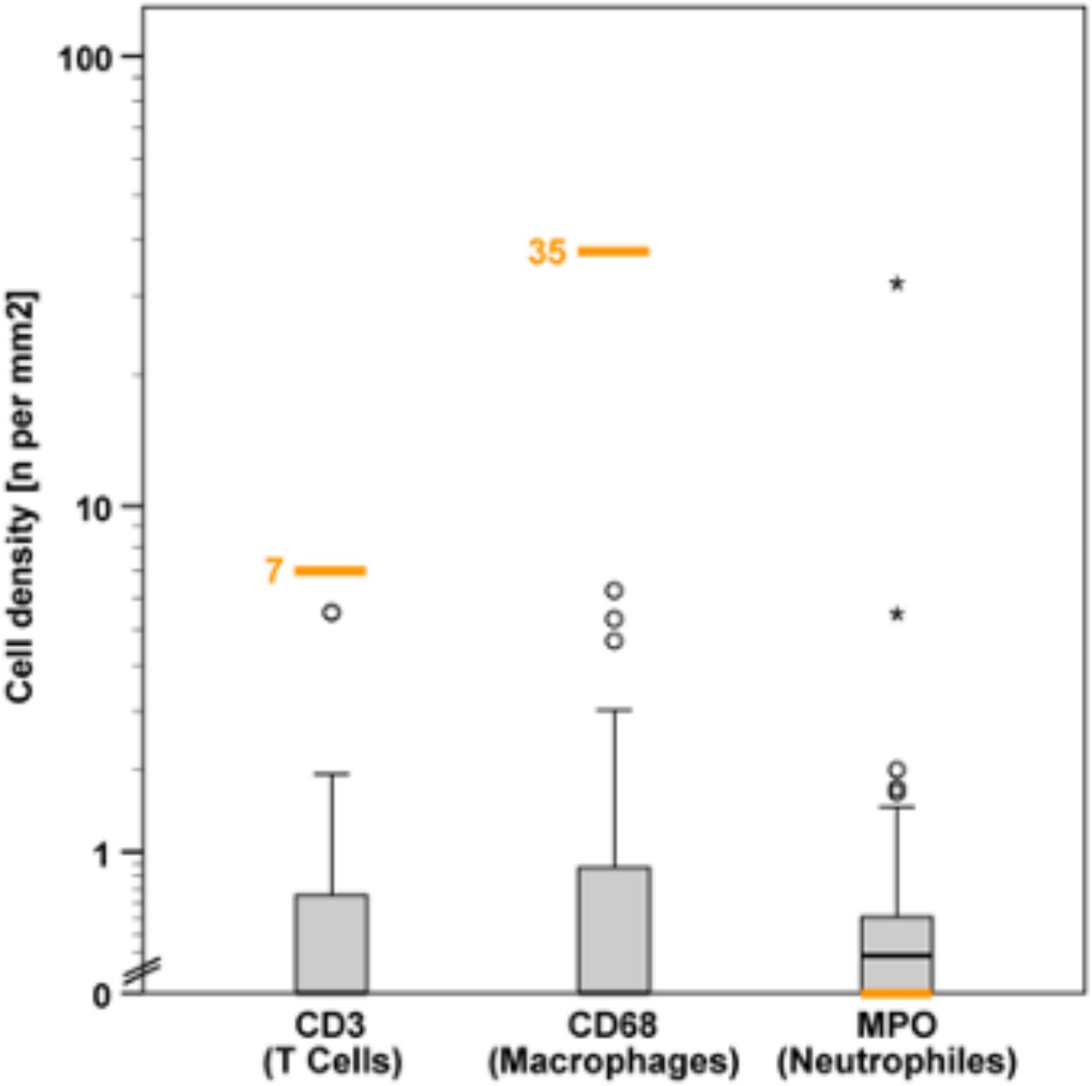
Density values of inflammatory cells in the myocardium of cardiac amyloidosis. Graphical representation of the distribution of density values of T lymphocytes (CD3-positive cells), macrophages (CD-68-positive cells), and neutrophiles (MPO-positive cells) on a logarithmic Y-axis, with corresponding threshold values marked (orange line), which represent the limits for inflammation (orange numbers) (Dominguez et al., 2016). The threshold value for neutrophils was depicted at 0. Values represented individually as circles or asterisks denote outliers or extreme values.

The present study aimed at analyzing the immune cell population in endomyocardial tissue samples of patients with cardiac amyloidosis and correlate it with cardiomyocyte diameter, necrosis, apoptosis and clinical parameters. The histological diagnosis of myocarditis is based on the application of the Dallas criteria, i.e., presence of inflammatory infiltrates in the myocardium associated with myocyte degeneration and necrosis of nonischemic cause^26^. According to Dominguez et al.^27^, inflammation is present when > 7.0 CD3 positive lymphocytes and > 35.0 macrophages/mm^2^ are detected. However, except for single cell necrosis, no case of our series harbored inflammatory cell densities beyond the cut-off values for T-cells and macrophages proposed by Dominguez et al.^27^ (Fig. 3). Thus, cardiac AL- and ATTR amyloidosis do not induce myocarditis. However, this does not preclude a pathophysiological role. In support of this contention, our results show that the number of CD68 positive cells correlated with NYHA class.

Nicolas-Avila wrote about cardioresident macrophages, which, alongside the dominating cardiomyocytes and fibroblasts, are an integral part of the cellular composition of cardiac tissue and play a pivotal role in myocardial tissue homeostasis^28^. Thus, in histological examinations of cardiac tissue, we should assume a corresponding number of CD68 positive cells, which is generally present in healthy cardiac muscle tissue. The fact that the number of CD68 positive cell infiltrations in the myocardium was significantly higher in advanced NYHA class indicates that macrophages are in fact involved in disease pathology^28^. The presence of CD68 positive macrophages has been described previously^29–32^. Stats and Stone (2016) assessed myocardial biopsies of ATTR and AL patients immunohistochemically and found CD68 positive cells in both amyloid types, albeit with a significantly higher density observed in AL compared to ATTR samples^33^. This is in contrast with findings of our study, where no significant correlation between CD68 positive cell density and amyloid type was determined. These differences might be explained by the low number of cases assessed by Stats and Stone, or the usage of different immunostaining protocols^33^. Macrophages might be involved in phagocytosis of amyloid fibrils or a cellular response to myocardial stress, yet the precise sequence of events is still unknown^34^. The immune cell infiltration is employed in treatment strategies using TTR antibodies that induce phagocytosis of ATTR aggregates and hence reduce amyloid load in the heart^35^.

A recent study by Sigismund et al. demonstrated that myocardial inflammation, specifically involving CD3 + and LFA1 + lymphocytes, predicts adverse outcomes in patients with AL amyloidosis^36^. Our initial focus was on examining the correlation between immune cell infiltration and clinical features in cardiac amyloidosis, emphasizing the role of CD3 + T lymphocytes, CD68 + macrophages, and MPO + neutrophils. Our results showed that while there was a significant correlation between cardiac macrophage density and heart failure, we found no significant correlation between the density of CD3 + T lymphocytes and patient outcomes. This contrasts with the findings of Sigismund et al., which indicated a predictive value for CD3 + and LFA1 + lymphocytes in AL amyloidosis^36^. The discrepancy could be due to several factors, including differences in patient cohorts, disease stages, or methodologies used in assessing immune cell infiltration.

To further investigate the implications of these contrasting results, future studies should focus on a more detailed analysis of immune cell subtypes, exploring whether specific subtypes like LFA1 + lymphocytes hold predictive value in cardiac amyloidosis outcomes. It is also important to consider the impact of patient demographics, treatment regimens, and other clinical variables that might influence the role of inflammation in the disease progression.

Kourelis et al. examined differences in the abundance of amyloid-specific proteins in relation to clinical patient characteristics. In contrast to AL amyloid deposits, an increase in complement proteins could be observed in ATTR amyloid-affected tissue^37^. Although the proteins were identified by laser microdissection and subsequent liquid chromatography, the results agree with ours. ATTR cases had a higher C9-stained biopsy area than AL cases. Amyloid type did not correlate with immune cell density, cardiomyocyte diameter, or apoptosis. This is in contrast to the results of Pucci et al., who assessed differences between cardiac tissues of both amyloid types and found significant differences between AL and ATTR in terms of amyloid load and fibrosis and the density of CD3 positive cells^32^. The reason for these differences is unknown but could be related to differences in the patient population or methodological differences in the quantification of immune cells. A significant correlation between amyloid type and C9 was observed. C9 is used as a surrogate marker for cardiomyocyte necrosis^38^. In a previous study, Lux et al. already described the presence of C9 in amyloid deposits, which was confirmed here in relation to inflammatory cells, and also noted the less frequent presence of Cas3^29^. Other studies corroborate the presence of C9 in amyloid deposits of ATTR tissues and other amyloidoses^37,39^. These findings, together with our results, point to a differential activation of the complement system in cardiac amyloidosis.

Our study has several limitations. Firstly, the results are based solely on immunohistochemical data. Ideally, immunohistochemical studies should be supported by additional experiments, particularly in terms of further characterizing the immune cell subpopulation and quantifying cell numbers. For example, fluorescent-assisted cell sorting could help to co-label immune cells with several different markers concomitantly and thereby determine subpopulations by the presence of multiple CD proteins. However, these studies require fresh tissue, and due to the nature and frequency of the disease, such tissue is difficult to obtain. Secondly, immunohistochemical studies provide no data regarding pathophysiological mechanisms leading to apoptosis and necrosis. In vitro studies using isolated myocytes may help to elucidate these mechanisms.

Furthermore, the unavailability of detailed genetic information precluded our ability to distinctly classify our study cohort into wild-type and hereditary ATTR amyloidosis subgroups, as well as between ALκ and ALλ subgroups. It should be noted that due to the loss of sections during processing and the finite availability of sample tissues, not all markers could be serially sectioned, and it was not feasible to repeat the procedure for those missing sections. We also acknowledge that while our study primarily utilized right ventricular biopsies, the representativeness of these samples for the entire heart, despite supporting literature, may still be subject to sampling error^17^.

In summary, our study provides further evidence of the putative pathophysiological role of inflammatory cells in cardiac amyloidosis. While the type and amount of amyloid did not correlate with immune cell densities, clinical disease parameters such as NYHA and NTproBNP correlated with macrophage density pointing towards a pathophysiological role of macrophages in cardiac amyloidosis.

## Electronic supplementary material

Below is the link to the electronic supplementary material.

Supplementary Material 1

## Data Availability

The datasets analysed during the current study are available from the corresponding author on reasonable request.

## Abbreviations

AL: Light chain amyloidosis
ATTR: Transthyretin amyloidosis
CD3: Cluster of differentiation 3
CD68: Cluster of differentiation 68
C9: Complement 9
Cas3: Caspase 3
FFPE: Formalin-fixed paraffin-embedded
H&E: Hematoxylin and eosin
LFA1+: Lymphocyte function-associated antigen 1
NYHA: New York Heart Association
