# Supplementary material for: Myocardial inflammatory cells in cardiac amyloidosis
Source: Sci Rep. 2024 Oct 7;14:23313. doi: 10.1038/s41598-024-74289-5 (PMC11458899; doi:10.1038/s41598-024-74289-5)
Supplement: Supplementary file 1 — Supplementary Material 1 [file 41598_2024_74289_MOESM1_ESM.docx]

**Supplementary file**

**Material and methods**

***Histology***

All samples had been fixed in formalin and embedded in paraffin (FFPE). Serial sections were cut from each paraffin block and stained with hematoxylin and eosin (H&E) and Congo red. The presence of amyloid was confirmed when a typical green-yellow-orange birefringence was found in cross-polarized light in Congo red stained tissue sections using a polarization microscope (Nikon ECLIPSE Ci POL; Hamburg, Germany). All examined specimens were obtained from amyloid bearing cardiac tissue.

***Immunohistochemistry***

Immunohistochemistry was carried out with commercially available monoclonal antibodies directed against complement 9 (C9; 1:400; Biozol, Eching, Germany), CD68 (1:100; Leica Biosystems, Wetzlar, Germany) and monoclonal rabbit antibodies directed against caspase 3 (Cas3; 1:100; Cell Signaling, Danvers, MA) and polyclonal rabbit antibodies directed against anti-lambda-light chain peptides (AL7, 1:200; Pineda, Berlin, Germany), transthyrethin (TTR3, 1:2000; Pineda, Berlin, Germany), CD3 (1:100; Neomarkers, Portsmouth, NH), myeloperoxidase (MPO; 1:100; DAKO, Hamburg, Germany). Immunostaining was done on FFPE sections with the Bond Max Leica immunostainer using the Bond Polymer Refine Detection Kit Leica Biosystems, Wetzlar, Germany). Antigen retrieval was carried out with Leica ER1-Bond Epitope Retrieval Solution 1 (C9), Leica ER2-Bond Epitope Retrieval Solution 2 (TTR3, caspase 3), Enzyme 1 (AL7; all Leica Biosystems, Wetzlar, Germany) and pronase E digestion and backing (C9) according to manufacturer´s instructions. Immunohistochemical classification of amyloid was carried out and had been validated as described elsewhere [22, 23].
In brief, identification of the amyloid was considered to be positive when there was a strong and homogenous immunostaining of the entire amyloid deposits. Uneven and weak staining of some deposits was not assumed to be proof of the amyloid protein.
